# Sirukumab in rheumatoid arthritis refractory to sulfasalazine or methotrexate: a randomized phase 3 safety and efficacy study in Japanese patients

**DOI:** 10.1186/s13075-018-1536-9

**Published:** 2018-03-07

**Authors:** Tsutomu Takeuchi, Hisashi Yamanaka, Masayoshi Harigai, Ryo Tamamura, Yuichi Kato, Yoshifumi Ukyo, Toshikazu Nakano, Benjamin Hsu, Yoshiya Tanaka

**Affiliations:** 1 Keio University School of Medicine, Shinjuku-ku, Tokyo, Japan; 2 Tokyo Women’s Medical University, Shinjuku-ku, Tokyo, Japan; 3 Janssen Pharmaceutical K.K., Chiyoda-ku, Tokyo, Japan; 4 Janssen Research & Development, LLC, Spring House, PA USA; 5 University of Occupational & Environmental Health Japan, Kitakyushu, Japan

**Keywords:** Biologicals, Disease-modifying anti-rheumatic drugs, Interleukin-6, Rheumatoid arthritis, Sirukumab

## Abstract

**Background:**

Sirukumab, a high-affinity human monoclonal antibody that selectively binds to interleukin-6, has demonstrated efficacy in the treatment of rheumatoid arthritis (RA) in global phase 1 and phase 2 studies. The present study evaluated the safety and efficacy of sirukumab, as monotherapy in Japanese patients with RA refractory to methotrexate or sulfasalazine.

**Methods:**

In this phase 3, double-blind study, 122 patients (age ≥ 20 years) were randomized (1:1, 61 patients in each arm) to sirukumab administered subcutaneously: 50 mg once every 4 weeks (q4w) or 100 mg once every 2 weeks (q2w) through 52 weeks. Disease-modifying anti-rheumatic drugs were allowed after 24 weeks. Safety was assessed and efficacy was evaluated using American College of Rheumatology (ACR) responses, Disease Activity Score C-reactive protein (DAS28-CRP) and Health Assessment Questionnaire-Disability Index (HAQ-DI).

**Results:**

Amongst the 122 randomized patients, 99 (81.1%) patients completed the study. Adverse events (AEs) were reported in 114/122 (93.4%) patients and serious AEs were reported in 9/122 (7.4%) patients. No deaths, major cardiovascular AEs, serious gastrointestinal perforations or tuberculosis cases were reported during this study period. Grade 3 hematologic abnormalities (neutropenia and leukopenia) were reported in seven patients and no grade 4 abnormalities were observed. ACR20 responses were observed within 2 weeks, achieved in 47/61 (77.0%, 50 mg q4w) patients and 44/61 (72.1%, 100 mg q2w) patients at week 16 and maintained through week 52. ACR50/70, DAS28-CRP and HAQ-DI responses were also maintained through week 52 in both groups.

**Conclusions:**

Safety findings were comparable between the two treatment groups. The 52-week administration of sirukumab at 50 mg q4w and 100 mg q2w was generally tolerable and with measurable efficacy in Japanese patients with RA refractory to methotrexate and sulfasalazine.

**Trial registration:**

NCT01689532. Registered 18 September 2012.

## Background

Newer treatment options have been introduced for rheumatoid arthritis (RA) in the past decades and anti-tumor necrosis factor (TNF) alpha agents have been well established as the first-line biologic agent of choice [1]. Although these agents have been used successfully in patients who had failed methotrexate (MTX) therapy, unresponsiveness and tolerability concerns have limited their use. Therefore, there exists an unmet need for newer therapeutic options that will safely alleviate the signs and symptoms of RA in patients who are nonresponsive to anti-TNF agents [2].

Interleukin (IL)-6, a key player in the development of RA, is found at elevated levels in the serum and synovial fluid of RA patients [3]. Currently, tocilizumab and sarilumab, humanized antibodies targeting the IL-6 receptor, are the only approved anti-IL-6 therapy for RA [4–6]. Sirukumab, an anti-IL-6 monoclonal antibody, binds selectively to IL-6 ligand with high affinity. In a phase 1 study, sirukumab demonstrated an acceptable safety and pharmacokinetic profile when administered intravenously (0.3–10 mg/kg) [7]. In a proof-of-concept phase 2 study, sirukumab administered subcutaneously (100 mg once in every 2 weeks) in combination with MTX demonstrated marked improvement (American College of Rheumatology 50 response [ACR50, ≥50% improvement] in 26.7% of patients at week 12) in patients with RA [8].

Patients with moderate to severe active RA refractory to MTX or sulfasalazine (SSZ) have a high unmet need and require strong immunomodulatory therapy to prevent further disease damage and morbidity. Additionally, optional therapies are needed in patients unable to tolerate disease-modifying anti-rheumatic drugs (DMARDs) such as MTX or SSZ due to toxicities such as liver functional failure [9, 10]. In a global phase 3 study (SIRROUND-D) conducted in active RA patients refractory to DMARDs (including MTX or SSZ), sirukumab showed significant improvements in RA symptoms along with inhibition of structural damage progression and improvements in quality of life [11]. Similar results were observed in another global 52-week phase 3 study in patients refractory or intolerant to anti-TNF therapies and other biological treatments (SIRROUND-T) [12]. Thus, sirukumab has demonstrated promise as a clinically efficacious therapy for RA treatment [13].

The current double-blind, randomized, phase 3 study was planned to evaluate the safety and efficacy of sirukumab monotherapy in Japanese patients with RA refractory to MTX or SSZ treatment. This is the first study to evaluate the benefits of sirukumab monotherapy in Japanese patients with active RA refractory to MTX or SSZ.

## Methods

### Patients

Japanese patients, age ≥ 20 years, diagnosed with RA (according to the revised 1987 criteria of the American Rheumatism Association) for ≥3 months before screening and unresponsive to MTX or SSZ, alone or in combination with other conventional synthetic disease -modifying anti-rheumatic drugs (csDMARDs), were enrolled. Patients with moderate to severely active RA with at least 6 of 68 tender joints and 6 of 66 swollen joints; positive for either rheumatoid factor or anti-cyclic citrullinated peptide or with baseline radiographic erosion; CRP level ≥ 0.8 mg/dl; and if using oral corticosteroids on a stable dose equivalent to ≤ 10 mg/day of prednisolone or on a stable dose of nonsteroidal anti-inflammatory drugs (NSAIDs) or analgesics for 2 weeks before first administration of sirukumab were included.

Patients with prior intolerance or inadequate response to biologic (b)DMARDs such as anti-TNF therapy or tocilizumab within 3 months of therapy, rituximab (other B-cell depleting therapy) within 7 months of therapy or prior sirukumab use were excluded from the study. Previous treatment with bDMARDs with discontinuation for other reasons (e.g., accessibility, inconvenience) was permitted. Other exclusion criteria were: patients who had received corticosteroids via intraarticular, intramuscular or intravenous route within 4 weeks prior to the first sirukumab administration; leflunomide in the past 24 months without any drug elimination procedure; cyclosporine A, azathioprine, tacrolimus, mycophenolate mofetil, oral or parenteral gold, or d-penicillamine within 4 weeks of sirukumab administration; and patients with uncontrolled or severe inflammatory disorders other than RA, hypersensitivity or intolerance to sirukumab, severe infections requiring hospitalization and chronic or recurring infectious disease.

The study protocol was approved by the institutional review board (Keio University Hospital) of the lead investigator (TT) and by the institutional review boards of each of the 20 other participating study sites. The study was conducted in accordance with the Declaration of Helsinki consistent with Good Clinical Practices and applicable regulatory requirements. All participants provided written informed consent.

### Study design, randomization and blinding

**Fig. 1:**
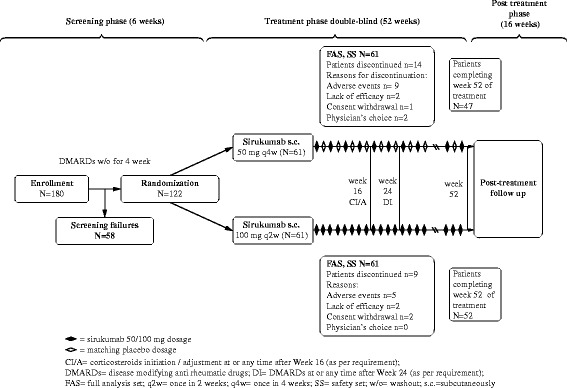
Study design and patient disposition

This phase 3, randomized, double-blind study, was conducted across 21 sites in Japan from October 2012 to March 2015. The study comprised three phases: screening phase (6 weeks), double-blind treatment phase (52 weeks) and posttreatment safety follow-up phase (16 weeks). After screening, the patients were randomized 1:1 to either sirukumab 50 mg every 4 weeks (50 mg q4w group) or sirukumab 100 mg every 2 weeks (100 mg q2w group) administered subcutaneously through week 52. Due to ethical considerations, a placebo group was not included. The two dose regimens were efficacious in the phase 2 study and were being evaluated in this and several phase 3 studies for a possible dose response. Placebo was administered at week 2 and thereafter every 4 weeks through week 52 to the 50 mg q4w group to maintain treatment blinding. Sirukumab (50 mg/1 ml or 100 mg/1 ml) or its placebo was supplied in a 1-ml prefilled syringe. The randomization was performed using a computer-generated randomization schedule and implemented using an interactive voice or web response system. Both patients and investigators were blinded until the safety follow-up was completed and the database finalized. Adjustment in NSAID or analgesic dosage was disallowed 2 weeks prior to first drug administration until week 16 of treatment. Patients with < 20% improvement in swollen or tender joints were allowed to initiate or adjust corticosteroid dosages at the investigator’s discretion from week 16 onward. Patients using csDMARDs at enrollment had to discontinue use 4 weeks prior to randomization and concomitant csDMARDS were not allowed until 24 weeks of sirukumab administration. From week 24 through week 52, patients with < 20% improvement from baseline in swollen and tender joints were allowed to take csDMARDs based on the investigator’s discretion (Fig. 1).

### Safety assessments

Safety (primary objective) was assessed by monitoring AEs, clinical laboratory tests, physical examinations, vital signs and chest X-ray scans for tuberculosis detection. The toxicity grade of clinical laboratory parameters was assessed by Common Terminology Criteria for Adverse Events (CTCAE) grading. The AEs were recorded by the patient’s voluntary reporting or their interview in an indirect manner at every visit. All serious AEs (SAEs), serious cardiovascular events, malignancies, infections such as tuberculosis, gastrointestinal perforations and clinical laboratory parameter abnormalities (hematologic, hepatobiliary and lipids) were monitored. Blood samples for clinical laboratory assessments were collected at baseline and weeks 2, 4, 6, 8, 12, 16, 18, 20, 24, 36, 44 and 52.

### Efficacy assessments

Efficacy variables included ACR responses and the Disease Activity Score using DAS28-CRP (responder, DAS28-CRP ≤ 3.2 to 5.1 at visit with 0.6 to > 1.2 improvement from baseline; remission, DAS28-CRP < 2.6). Major clinical response was achieved if the ACR70 response was sustained for 6 months. Improvements in disease activity were assessed by Simplified Disease Activity Index (SDAI) and Clinical Disease Activity Index (CDAI) responses. Treatment failures included patients who started treatment with csDMARDs, systemic immunosuppressives (e.g., leflunomide) and/or bDMARDs or required additional treatment or increased dose of oral corticosteroids, intravenous/intramuscular administration of corticosteroids, or discontinued sirukumab for any reason through week 52.

Additionally, physical function was assessed by the Health Assessment Questionnaire-Disability Index (HAQ-DI). Efficacy variables were assessed every 2 weeks from week 0 to 8, and thereafter every 4 weeks through week 52. Efficacy was also assessed up to 16 weeks in the posttreatment follow-up phase to analyze the effect of concomitant medication.

### Immunogenicity assessment

Immunogenicity was assessed from blood samples using a validated drug-tolerant enzyme immunoassay (EIA) at weeks 0, 24 and 52 and final safety follow-up visit (week 68).

### Statistical analysis

As per the Japanese regulatory authorities, ≥ 100 patients are recommended to be exposed to the study drug for 1 year to obtain the necessary safety data. Considering the number of Japanese patients in the other global studies of sirukumab, ≥ 40 patients per treatment group were required in this study. Additionally, assuming that about 70% of patients would complete 1 year of treatment as in a study of similar design [14], a minimum enrollment of 120 patients (60 per treatment group) was required.

The safety analysis set included patients who received ≥ 1 dose of sirukumab. The efficacy full analysis set included all patients randomized to the study. Data imputation by the nonresponder imputation (NRI) or the last observation carried forward (LOCF) method was used for categorical and continuous efficacy parameters, respectively. The treatment failure rule was applied to data for posttreatment efficacy analysis. No comparative statistical tests were performed. Descriptive statistics were provided for safety and efficacy parameters (mean and median values of data are provided based on parameter and data distribution).

## Results

### Patient disposition and characteristics

Of the 180 patients enrolled, 122 patients (50 mg q4w group, *N* = 61; 100 mg q2w group, *N* = 61) were randomized, treated and included in the safety and efficacy analysis. Among these, 99 (81.1%) patients completed the study, with the most common reason for study discontinuation being AEs (50 mg q4w group, 9/61 (14.8%) patients; 100 mg q2w group, 5/61 (8.2%) patients) (Fig. 1, Table 2); however, there was no specific trend in these AEs.

The baseline characteristics and demographics between both treatment groups were well balanced. The majority of patients were women (90/122 (73.8%)) with mean (SD) age of 55.1 (11.41) years and body mass index (BMI) 22.3 (3.58) kg/m^2^, and the median (range) duration of RA was 5.77 (0.4–41.0) years (Table 1). The baseline RA characteristics for ACR components were also balanced with median (range) CRP levels being 2.6 (0.2–16.6) mg/dl. The proportions of patients exposed to prior treatments include ≥ 1 DMARD 100%, ≥ 2 DMARDs 60.7%, MTX 97.5%, SSZ 36.1% and biologics other than sirukumab 19.7% (14 patients (23.0%) in 50 mg q4w group and 10 patients (16.4%) in 100 mg q2w group). The study excluded patients with previous intolerance or inadequate response to tumor necrosis factor (TNF) inhibitor or tocilizumab (anti-IL-6 receptor) therapy and use of B-cell depleting therapy within the previous 7 months. The median sirukumab treatment duration and treatment administrations were 52.14 weeks and 27, respectively. Only three patients (2/61 patients in 50 mg q4w group and 1/61 patient in 100 mg q2w group) initiated DMARDs for minimal improvement in swollen and tender joints at or any time after week 24.

**Table 1:** Patient demographics and baseline characteristics (efficacy full analysis set)

Characteristic	Sirukumab 50 mg q4w (*N* = 61)	Sirukumab 100 mg q2w (*N* = 61)	All (*N* = 122)
Sex (women), *n* (%)	47.0 (77.0)	43.0 (70.5)	90 (73.8)
Age (years), mean (SD)	55.4 (10.70)	54.7 (12.16)	55.1 (11.41)
Weight (kg), mean (SD)	55.0 (12.23)	56.8 (9.71)	55.9 (11.03)
Height (cm), mean (SD)	157.9 (7.90)	158.4 (7.80)	158.1 (7.82)
BMI (kg/m^2^), mean (SD)	22.0 (3.98)	22.6 (3.13)	22.3 (3.58)
Disease duration (years), median (range)	5.0 (0.4–41.0)	6.3 (0.4–30.0)	5.8 (0.4–41.0)
Swollen joints (number 0–66), median (range)	11.0 (6.0–22.0)	11.0 (5.0–32.0)	11.0 (5.0–32.0)
Tender joints (number 0–68), median (range)	14.0 (6.0–56.0)	15.0 (5.0–48.0)	14.0 (5.0–56.0)
Patient’s assessment of pain (VAS; 0–10 cm), median (range)	7.4 (0.7–10.0)	7.4 (0.5–10.0)	7.4 (0.5–10.0)
Patient’s global assessment of disease activity (VAS; 0–10 cm), median (range)	7.6 (0.6–10.0)	7.1 (0.6–10.0)	7.5 (0.6–10.0)
Physician’s global assessment of disease activity (VAS; 0–10 cm), median (range)	6.5 (2.6–10.0)	6.9 (2.0–10.0)	6.6 (2.0–10.0)
HAQ-DI score (0–3), median (range)	1.4 (0.00–2.75)	1.1 (0.00–2.75)	1.3 (0.00–2.75)
CRP (mg/dl), median (range)	2.7 (0.20– 9.20)	2.5 (0.31–16.60)	2.6 (0.20–16.60)
DAS28-CRP score, median (range)	5.6 (5.14–6.11)	5.9 (4.88–6.57)	5.7 (4.93–6.29)
SDAI score, median (range)	33.2 (28.42–40.38)	38.2 (26.83–52.20)	34.7 (27.70–42.48)
CDAI score, median (range)	30.9 (24.90–37.80)	33.5 (24.20–47.40)	31.8 (24.60–38.80)
Rheumatoid factor-positive, *n* (%)	48 (78.7)	44 (72.1)	92 (75.4)
Anti-CCP antibody positive, *n* (%)	55 (90.2)	51 (83.6)	106 (86.9)

*BMI* body mass index, *CCP* cyclic citrullinated peptide, *CDAI* Clinical Disease Activity Index, *CRP* C-reactive protein, *HAQ-DI* Health Assessment Questionnaire-Disability Index, *q4w* once in 4 weeks, *q2w* once in 2 weeks, *SDAI* Simplified Disease Activity Index, *SD* standard deviation, *VAS* visual analog scale

### Safety

#### Adverse events

Of the 122 patients in the safety set, 114 (93.4%) patients experienced ≥ 1 AE and this was comparable between the two treatment groups (50 mg q4w group, 56/61 (91.8%) patients; 100 mg q2w group, 58/61 (95.1%) patients). The most common AEs were nasopharyngitis, injection-site erythema and injection-site swelling (Table 2). Among AEs of special interest, infections were observed in 77/122 (63.1%) patients and injection-site reactions were observed in 47/122 (38.5%) patients.

**Table 2:** Summary of adverse events through week 52 (safety analysis set)

	Sirukumab 50 mg q4w (*N* = 61)	Sirukumab 100 mg q2w (*N* = 61)	All (*N* = 122)
AEs	56 (91.8)	58 (95.1)	114 (93.4)
Serious AEs	4 (6.6)	5 (8.2)	9 (7.4)
AEs in ≥ 10% patients			
Infections and infestations	39 (63.9)	38 (62.3)	77 (63.1)
Nasopharyngitis	27 (44.3)	27 (44.3)	54 (44.3)
Pharyngitis	6 (9.8)	7 (11.5)	13 (10.7)
General disorders and administration site conditions	26 (42.6)	27 (44.3)	53 (43.4)
Injection-site erythema	19 (31.1)	20 (32.8)	39 (32.0)
Injection-site swelling	10 (16.4)	12 (19.7)	22 (18.0)
Injection-site pruritus	7 (11.5)	13 (21.3)	20 (16.4)
Investigations	28 (45.9)	21 (34.4)	49 (40.2)
Alanine aminotransferase increased	10 (16.4)	10 (16.4)	20 (16.4)
Aspartate aminotransferase increased	9 (14.8)	11 (18.0)	20 (16.4)
White blood cell count decreased	7 (11.5)	7 (11.5)	14 (11.5)
Platelet count decreased	9 (14.8)	3 (4.9)	12 (9.8)
Neutrophil count decreased	7 (11.5)	4 (6.6)	11 (9.0)
Skin and subcutaneous tissue disorders	15 (24.6)	25 (41.0)	40 (32.8)
Eczema	7 (11.5)	7 (11.5)	14 (11.5)
Vascular disorders	5 (8.2)	7 (11.5)	12 (9.8)
Hypertension	5 (8.2)	7 (11.5)	12 (9.8)
Serious AEs			
Infections and infestations	1 (1.6)	2 (3.3)	3 (2.5)
Acute sinusitis	0	1 (1.6)	1 (0.8)
Hepatitis E	0	1 (1.6)	1 (0.8)
Osteomyelitis	1 (1.6)	0	1 (0.8)
Gastrointestinal disorders	1 (1.6)	1 (1.6)	2 (1.6)
Gastroesophageal reflux disease	0	1 (1.6)	1 (0.8)
Large intestine polyp	1 (1.6)	0	1 (0.8)
Injury, poisoning and procedural complications			
Comminuted fracture	1 (1.6)	0	1 (0.8)
Musculoskeletal and connective tissue disorders			
Intervertebral disc protrusion	0	1 (1.6)	1 (0.8)
Neoplasms benign, malignant and unspecified			
Borderline serous tumor of ovary	0	1 (1.6)	1 (0.8)
Psychiatric disorders			
Schizophrenia	1 (1.6)	0	1 (0.8)
Reproductive system and breast disorders			
Endometriosis	0	1 (1.6)	1 (0.8)

*Data presented as n* (%)

*AE* adverse event, *q2w* once in 2 weeks, *q4w* once in 4 weeks

#### Serious adverse events

Overall, 9/122 (7.4%) patients had ≥ 1 SAE (50 mg q4w group, 4/61 (6.6%) patients; 100 mg q2w group, 5/61 (8.2%) patients) (Table 2). Serious infections of sinusitis and hepatitis E (one patient each in 100 mg q2w group, 2/61 (3.3%)) and osteomyelitis (50 mg q4w group, 1/61 (1.6%) patient) were observed. Gastrointestinal disorders occurred in one patient each in both groups. Comminuted fracture and schizophrenia (one patient each) were reported in the 50 mg q4w group whereas endometriosis with borderline serous ovary tumor and intervertebral disc protrusion (one patient each) was reported in the 100 mg q2w group. No major cardiovascular events, tuberculosis or gastrointestinal perforations were observed (Table 2). After completion of the posttreatment follow-up period, one patient reported myocardial infarction but the patient recovered from the event. No deaths were observed during the 52-week study treatment.

#### Hematology and clinical laboratory values

Patients who had grade 0 hematology laboratory values at baseline reported shifts to grade 3 events through week 52 with respect to decreased neutrophils (50 mg q4w group, 3/61 patients; 100 mg q2w group, 2/61 patients) and decreased leukocytes (100 mg q2w group, 2/61 patients) (Table 3). A grade 4 event of decreased platelets (100 mg q2w group, 1/61 patient) was also reported but was considered a technical error, and no events of associated bleeding were reported. Occurrence of severe infection was not observed in patients with grade 3 neutropenia.

**Table 3:** Summary of safety events related to blood parameters and hematology: shift from CTCAE grade 0 at baseline through week 52 (safety analysis set)

Parameter	Total (*N*)	Grade 0 at baseline	Grade 0	Grade 1	Grade 2	Grade 3	Grade 4
Neutrophils decreased							
Sirukumab 50 mg q4w	61	61 (100.0)	33 (54.1)	17 (27.9)	8 (13.1)	3 (4.9)	0
Sirukumab 100 mg q2w	61	61 (100.0)	34 (55.7)	14 (23.0)	11 (18.0)	2 (3.3)	0
Platelets decreased							
Sirukumab 50 mg q4w	61	61 (100.0)	44 (72.1)	17 (27.9)	0	0	0
Sirukumab 100 mg q2w	61	61 (100.0)	54 (88.5)	6 (9.8)	0	0	1 (1.6)^a^
Leukocytes decreased							
Sirukumab 50 mg q4w	61	61 (100.0)	34 (55.7)	21 (34.4)	6 (9.8)	0	0
Sirukumab 100 mg q2w	61	61 (100.0)	42 (68.9)	11 (18.0)	6 (9.8)	2 (3.3)	0
Lymphocytes decreased							
Sirukumab 50 mg q4w	61	58 (95.1)	51 (83.6)	3 (4.9)	4 (6.6)	0	0
Sirukumab 100 mg q2w	61	58 (95.1)	50 (82.0)	1 (1.6)	7 (11.5)	0	0
Alanine aminotransferase increased							
Sirukumab 50 mg q4w	61	58 (95.1)	30 (49.2)	26 (42.6)	2(3.3)	0	0
Sirukumab 100 mg q2w	61	56 (91.8)	31 (50.8)	24 (39.3)	1 (1.6)	0	0
Aspartate aminotransferase increased							
Sirukumab 50 mg q4w	61	59 (96.7)	33 (54.1)	25 (41.0)	1 (1.6)	0	0
Sirukumab 100 mg q2w	61	55 (90.2)	32 (52.5)	23 (37.7)	0	0	0
Bilirubin increased							
Sirukumab 50 mg q4w	61	61 (100.0)	48 (78.7)	10 (16.4)	3 (4.9)	0	0
Sirukumab 100 mg q2w	61	61 (100.0)	47 (77.0)	10 (16.4)	4 (6.6)	0	0
Cholesterol increased							
Sirukumab 50 mg q4w	59	59 (100.0)	51 (83.6)	5 (8.2)	3 (4.9)	0	0
Sirukumab 100 mg q2w	60	60 (100.0)	54 (88.5)	5 (8.2)	1 (1.6)	0	0
Triglycerides increased							
Sirukumab 50 mg q4w	59	55 (93.2)	34 (55.7)	20 (32.8)	0	1 (1.6)	0
Sirukumab 100 mg q2w	60	54 (90.0)	33 (54.1)	20 (32.8)	0	1 (1.6)	0

*Data presented as n* (%) unless stated otherwise

*CTCAE* Common Terminology Criteria For Adverse Events, *q4w* once in 4 weeks, *q2w* once in 2 weeks

^a^Erroneously reported

Hepatobiliary parameters (aspartate transaminase (AST), alanine transaminase (ALT) and bilirubin) that were grade 0 at baseline did not demonstrate grade 3 or 4 shifts through week 52. Few events of grade 2 shifts (0 at baseline) in ALT (*n* = 3) and AST (*n* = 1) were observed, which returned to normal at week 52. In all, seven patients had a grade 2 shift in bilirubin but without any associated increases in ALT or AST. The majority of lipid abnormalities (increased serum triglyceride and cholesterol) were grade 1 or 2 in severity. A grade 3 serum triglyceride increase was observed in two patients (1.7%) and the values remained high even at week 52 (Table 3). Overall, there was no dose-dependent effect on the safety profile of sirukumab with respect to clinical laboratory parameters.

### Efficacy

The ACR20/50/70 responses at week 16 were achieved in 77.0%, 47.5% and 26.2% of patients in the 50 mg q4w group and in 72.1%, 57.4% and 32.8% of patients in the 100 mg q2w group, and were maintained through week 52 (Table 4). ACR50/70 responses in the 100 mg q2w group were numerically higher versus the 50 mg q4w group. Major clinical response was achieved by 23/122 (18.9%) patients (50 mg q4w group, 8/61 (13.1%) patients; 100 mg q2w group, 15/61 (24.6%) patients). Overall, subgroup analysis demonstrated that demographics, baseline disease characteristics and medication use did not have any effect on achieving ACR20 at week 16. ACR20 in patients with prior biologic use was 10/17 (58.8%) patients in the 50 mg q4w group and 11/13 (84.6%) patients in the 100 mg q2w group at week 52.

**Table 4:** Summary of efficacy parameters through week 52 (efficacy full analysis set^a^)

Efficacy parameter	Sirukumab 50 mg q4w (*N* = 61)	Sirukumab 100 mg q2w (*N* = 61)
ACR20 response, *n* (%)		
Week 16	47 (77.0)	44 (72.1)
Week 24	45 (73.8)	50 (82.0)
Week 52	39 (63.9)	46 (75.4)
ACR50 response, *n* (%)		
Week 16	29 (47.5)	35 (57.4)
Week 24	30 (49.2)	39 (63.9)
Week 52	29 (47.5)	35 (57.4)
ACR70 response, *n* (%)		
Week 16	16 (26.2)	20 (32.8)
Week 24	15 (24.6)	22 (36.1)
Week 52	20 (32.8)	24 (39.3)
HAQ-DI		
Week 16 median decrease from baseline, %	41.2	51.5
Week 52 median decrease from baseline, %	44.5	70.0
CRP levels (mg/dl), mean (SD)		
Week 16	0.02 (0.05)	0.02 (0.02)
Week 52	0.02 (0.06)	0.02 (0.02)
DAS28-CRP response		
Week 16 responders, *n* (%)	55 (90.2)	59 (96.7)
Week 52 responders, *n* (%)	45 (73.8)	50 (82.0)
DAS28-CRP remission		
Week 16 remitters, *n* (%)	28 (45.9)	30 (49.2)
Week 52 remitters, *n* (%)	29 (47.5)	32 (52.5)
SDAI		
Week 16 change from baseline, mean (SD)	−22.4 (12.44)	−25.9 (13.99)
Week 52 change from baseline, mean (SD)	−23.3 (14.02)	−28.5 (15.94)
CDAI		
Week 16 change from baseline, mean (SD)	−19.4 (12.14)	−22.7 (13.23)
Week 52 change from baseline, mean (SD)	−20.4 (13.83)	−25.3 (15.26)
HAQ-DI response		
Week 16 responders, *n* (%)	46 (75.4)	41 (67.2)
Week 52 responders, *n* (%)	44 (72.1)	41 (67.2)

*ACR20/50/70* American College of Rheumatology 20/50/70 response, *CDAI* Clinical Disease Activity Index, *CRP* C-reactive protein, *DAS28* Disease Activity Score 28, *HAQ-DI* Health Assessment Questionnaire-Disability Index, *SD* standard deviation, *SDAI* Simplified Disease Activity Index, *q4w* once in 4 weeks, *q2w* once in 2 weeks

^a^Data imputation was nonresponder imputation for categorical parameters and last observation carried forward for noncategorical parameters

Improvements in HAQ-DI were observed early in the study (50 mg q4w group, week 2; 100 mg q2w group, week 4). The HAQ-DI response (decrease in score ≥ 0.22) was observed in 75.4% patients (50 mg q4w group) and 67.2% patients (100 mg q2w group) at week 16 and the response rates were maintained through week 52 (Table 4). The CRP concentration was almost undetectable from week 16 through week 52. The maximum DAS28-CRP responders were observed at week 4 (58/61 (95.1%) patients in each group), which decreased slightly to 45/61 (73.8%) responders in the 50 mg q4w group and 50/61 (81.9%) responders in the 100 mg q2w group at week 52 (Table 4). The percentage of patients achieving DAS28-CRP remission (< 2.6) increased over time and maximum remission rates were achieved at week 24 (50 mg q4w group, 30/61 (49.2%) patients; 100 mg q2w group, 36/61 (59.0%) patients) and were generally maintained at week 52 (50 mg q4w group, 29/61 (47.5%) patients; 100 mg q2w group, 32/61 (52.5%) patients) (Table 4). In patients with prior bDMARD use, the DAS28-CRP remission rates at week 52 were 41.2% (7/17) patients in the 50 mg q4w group and 76.9% (10/13) patients in the 100 mg q2w group. The SDAI and CDAI assessments showed an improvement at week 16, which was consistent until week 52 (Table 4).

#### Efficacy assessments post sirukumab administration

DMARDs were initiated after the last sirukumab dose at week 52, except in three patients (50 mg q4w group, 2/61 patients; 100 mg q2w group, 1/61 patient) who received DMARDs on or post week 24 (< 20% improvement in swollen and tender joint counts). Proportions of patients with ACR20 and ACR50 response at week 16 post sirukumab treatment were generally similar as compared with week 52 (end of treatment) regardless of DMARDs added (Table 5). In patients using concomitant MTX or corticosteroids (oral or intramuscular), the mean DAS28-CRP was ≤ 2.6 for the 100 mg q2w group at week 52 (Table 5).

**Table 5:** Summary of ACR20 and ACR50 responses and DAS28-CRP based on concomitant medication at and after week 52 (efficacy full analysis set)

Efficacy parameter	Sirukumab 50 mg q4w	Sirukumab 100 mg q2w
ACR20 response		
Methotrexate		
Week 52^a^	29/44 (65.9)	34/42 (81.0)
Week 52^b^	29/36 (80.6)	34/38 (89.5)
Week 16, posttreatment phase^b^	32/36 (88.9)	32/38 (84.2)
DMARDs (including methotrexate)		
Week 52^a^	31/49 (63.3)	34/44 (77.3)
Week 52^b^	31/38 (81.6)	34/38 (89.5)
Week 16, posttreatment phase^b^	33/38 (86.8)	32/38 (84.2)
Corticosteroid (oral)		
Week 52^a^	2/7 (28.6)	8/11 (72.7)
Week 52^b^	2/3 (66.7)	8/9 (88.9)
Week 16, posttreatment phase^b^	3/3 (100.0)	9/9 (100.0)
Corticosteroid (oral, intramuscular)		
Week 52^a^	4/11 (36.4)	11/15 (73.3)
Week 52^b^	4/6 (66.7)	11/12 (91.7)
Week 16, posttreatment phase^b^	5/6 (83.3)	12/12 (100.0)
No DMARD and corticosteroid		
Week 52^a^	7/11 (63.6)	8/12 (66.7)
Week 52^b^	8/8 (100.0)	8/10 (80.0)
Week 16, posttreatment phase^b^	8/8 (100.0)	8/10 (80.0)
ACR50 response		
Methotrexate		
Week 52^a^	21/44 (47.7)	24/42 (57.1)
Week 52^b^	21/36 (58.3)	24/38 (63.2)
Week 16, posttreatment phase^b^	23/36 (63.9)	27/38 (71.1)
DMARDs (including methotrexate)		
Week 52^a^	22/49 (44.9)	24/44 (54.5)
Week 52^b^	22/38 (57.9)	24/38 (63.2)
Week 16, posttreatment phase^b^	23/38 (60.5)	27/38 (71.1)
Corticosteroid (oral)		
Week 52^a^	0/7 (0)	6/11 (54.5)
Week 52^b^	0/3 (0)	6/9 (66.7)
Week 16, posttreatment phase^b^	1/3 (33.3)	8/9 (88.9)
Corticosteroid (oral, intramuscular)		
Week 52^a^	1/11 (9.1)	9/15 (60.0)
Week 52^b^	1/6 (16.7)	9/12 (75.0)
Week 16, posttreatment phase^b^	2/6 (33.3)	11/12 (91.7)
No DMARD and corticosteroid		
Week 52^a^	7/11 (63.6)	8/12 (66.7)
Week 52^b^	7/8 (87.5)	8/10 (80.0)
Week 16, posttreatment phase^b^	7/8 (87.5)	8/10 (80.0)
DAS28-CRP score		
All, *n*	61	61
Week 52,^b^ *n*	47	52
Mean (SD)	2.4 (1.07)	2.3 (1.03)
Week 16, posttreatment phase, *n*^b^	61	60
Mean (SD)	2.5 (1.17)	2.4 (1.19)
Methotrexate, *n*	44	42
Week 52, *n*^b^	36	38
Mean (SD)	2.5 (1.09)	2.5 (0.94)
Week 16, posttreatment phase, *n*^b^	44	41
Mean (SD)	2.5 (1.10)	2.6 (1.18)
Corticosteroid (oral, intramuscular), *n*	11	15
Week 52, *n*^b^	6	12
Mean (SD)	3.0 (1.69)	1.9 (0.97)
Week 16, posttreatment phase, *n*^b^	11	15
Mean (SD)	3.0 (1.59)	2.1 (0.89)

Data presented as *n*/*N* (%)

^a^Data imputation was nonresponder imputation for categorical parameters and last observation carried forward for noncategorical parameters

^b^Week 52 and week 16, posttreatment phase: values were as observed

*ACR20/50/70* American College of Rheumatology 20/50/70 response, *DMARD* Disease-modifying anti-rheumatic drug, *n* number of patients, *N* total number of patients, *q4w* once in 4 weeks, *q2w* once in 2 weeks, *SD* standard deviation

#### Immunogenicity

Only patients in the 50 mg q4w group (6/61 patients) were positive for antibodies against sirukumab through week 68. In two of these patients, immunogenicity was associated with injection-site reactions. No patients were positive for neutralizing antibodies.

## Discussion

A 52-week exposure to sirukumab monotherapy (50 mg q4w and 100 mg q2w) was generally tolerable in Japanese patients with active RA who were nonresponders to MTX or SSZ. A retention rate of 81.1% suggests that sirukumab was tolerable, safe and efficacious over 52 weeks of therapy.

Infections related to the immunosuppression were the most commonly observed AEs in anti-IL-6 therapies. The phase 2 trials of sirukumab (100 mg q2w for 24 weeks, 36.7% of patients), sarilumab (200 mg q2w for 12 weeks, 23.5% of patients), tocilizumab (8 mg/kg infusion q4w for 24 weeks, 32.0% of patients) and olokizumab (120 mg q2w for 12 weeks, 27.3% of patients) reported comparable AEs of infections [6, 8, 15, 16]. In all studies, MTX was administered with IL-6 inhibitor, except for the olokizumab study. A phase 2b study in RA patients also reported 12 serious infections (of 28 SAEs) in clazakizumab-treated patients (with or without MTX) [17]. Further, the proportion of patients reporting injection-site reactions was comparable between both treatment groups (50 mg q4w group, 23/61 patients; 100 mg q2w group, 24/61 patients). An association between injection-site reaction and anti-sirukumab antibodies was reported in two patients (50 mg q4w group). Overall, the incidences of ≥ 1 AEs, infections and injection-site reactions were higher in this phase 3 study as compared to observations from a shorter 38-week, phase 2 study (100 mg q2w group: ≥ 1 AEs, 86.7%; infections, 36.7%; injection-site reaction, 16.7%) and two 52-week global phase 3 studies: SIRROUND-D (100 mg q2w group: ≥ 1 AEs, 80.2%; injection-site reactions, 16.3%) and SIRROUND-T (100 mg q2w group: ≥ 1 AEs, 81.0%; injection-site reactions, 25.0%) [8, 11, 12]. Discontinuations due to AEs in this study were primarily due to infections and infestations (4/122 patients) and the proportion of patients discontinuing treatment due to AEs was lower (3.3%) as compared with the SIRROUND-D (7.8%) and SIRROUND-T (10.1%) studies [11, 12].

The proportion of patients with ≥ 1 SAE was low (7.4%) and comparable with other anti-IL-6 therapy studies of similar treatment duration [4, 6, 16]. The SAEs observed in the earlier phase 2 sirukumab study were similar in profile and frequency to those in this study, suggesting that 52-week sirukumab administration did not significantly affect patient proportions with SAEs. Also, the proportion of patients with SAEs was lower as compared with the rates reported in SIRROUND-D (10.4%) and SIRROUND-T (13.7%) [11, 12]. Overall, three SAEs related to infections (acute sinusitis, hepatitis E and osteomyelitis in one patient each) were reported during the study (50 mg q4w group, 1 SAE; 100 mg q2w group, 2 SAEs). No deaths were reported in this study, although an imbalance in deaths and malignancy has been reported in the sirukumab group as compared with the placebo group in the global SIRROUND-D and SIRROUND-T studies [11, 12]. Incidences of major cardiovascular events, gastrointestinal perforations or tuberculosis have also been reported in these global studies; however, these were absent in the current study [11, 12]. Hematological abnormalities, a common feature noted in all anti-IL-6 therapy studies, were also observed in the current study but no grade 4 events were reported. No patients developed neutralizing antibodies to sirukumab.

Improvements in signs and symptoms of RA based on ACR20/50/70 responses, DAS28-CRP response and HAQ-DI score occurred as early as 2 weeks of treatment with sirukumab and were maintained or improved through week 52. These improvements were numerically greater in the100 mg q2w group versus the 50 mg q4w group. In this 52-week study, the proportion of patients achieving ACR20 responses at week 16 (100 mg q2w group, 72.1% patients) was consistent with the phase 2 study (100 mg q2w group, 63.3%) [8]. Efficacy in the phase 2 studies of sirukumab with respect to ACR20 was similar to those for sarilumab, olokizumab and clazakizumab [4, 6, 8, 15, 16, 18]. However, the ACR70 response at week 52 with both doses (50 mg q4w group, 32.8% patients; 100 mg q2w group, 39.3% patients) was reported in a higher percentage of patients as compared with the SIRROUND-D study (52 weeks; 50 mg q4w group, 16.5%; 100 mg q2w group, 18.5%) and the SIRROUND-T study (24 weeks; 50 mg q4w group, 9%; 100 mg q2w group, 10%) [11, 12]. Similarly, a higher percentage of patients achieved DAS28-CRP remission in the current study at week 52 (50 mg q4w group, 47.5%; 100 mg q2w group, 52.5%) as compared with the phase 2 study (24 weeks; 50 mg q4w group, 13.3%; 100 mg q2w group, 20.0%), the SIRROUND-D study (24 weeks; 50 mg q4w group, 26.0%; 100 mg q2w group, 25.5%) and the SIRROUND-T study (24 weeks; 50 mg q4w group, 19.0%; 100 mg q2w group, 22.0%) [8, 11, 12]. The suppression of inflammation as reflected by the consistently low levels of CRP (0.02 mg/dl from week 16 to 52) may confirm the ability of sirukumab to block IL-6 signaling during the span of the dosing interval [19]. Improvements in CDAI were also consistent with changes in other measures of disease activity (ACR and DAS28-CRP) and maximum improvement was observed at week 52. Concomitant MTX was started post week 52 in most of the patients and demonstrated maintenance of efficacy until week 16 post treatment in both treatment groups. In addition, some patients maintained ACR50 response without concomitant DMARDs and corticosteroids during the posttreatment phase. Overall, the efficacy profile of sirukumab monotherapy over a 52-week period in Japanese patients with active RA support findings from the global SIRROUND-D and SIRROUND-T studies [11, 12]. The current data in combination with previous randomized controlled trials of sirukumab suggest a potential role for sirukumab in RA therapy.

Limitations of this study include the low number of patients and lack of efficacy endpoints. The absence of a placebo control is a limitation for this study with respect to the absolute efficacy achieved but consistent improvement was observed in both sirukumab-treated groups in terms of the clinical signs and symptoms of RA. Generalizability and extrapolation of these results to routine clinical practice should be made within the context that the patients discontinued csDMARDs before study participation, in contrast to the recommended treatment modality that includes continued use of DMARDs for greater efficacy in patients with active RA [14, 20].

## Conclusions

Sirukumab monotherapy at both doses (50 mg q4w and 100 mg q2w) was generally tolerable and the safety profile was dosage independent in Japanese patients with RA refractory to MTX and SSZ in this 52-week study. Thus, sirukumab monotherapy has the potential to be a new viable treatment option for RA refractory to conventional synthetic or biologic DMARDs.
